# ‘Tell me what you know’: Written recall as a window into illness script quality

**DOI:** 10.1111/medu.70314

**Published:** 2026-09-17

**Authors:** Fernando Salvetti Valente, Ligia Maria Cayres Ribeiro, Andre Santanchè, Hassan Rahhal, Silvia Mamede, Pedro Tadao Hamamoto Filho, Marco Antonio de Carvalho Filho

**Affiliations:** ^1^ University Medical Centre Groningen University of Groningen Groningen The Netherlands; ^2^ Department of Internal Medicine University of São Paulo São Paulo Brazil; ^3^ Department of Information Systems State University of Campinas (UNICAMP) Campinas São Paulo Brazil; ^4^ Department of Internal Medicine of Hospital das Clínicas HCFMUSP, School of Medicine University of São Paulo (USP) São Paulo São Paulo Brazil; ^5^ School of Medicine University of São Caetano do Sul São Paulo São Paulo Brazil; ^6^ Department of Neurosciences and Mental Health of the São Paulo State University (UNESP) Botucatu São Paulo Brazil

## Abstract

**Introduction:**

Illness scripts (mental representations of disease knowledge) are central to diagnostic expertise, yet structured methods to examine their quality across medical training are lacking. This exploratory study investigated whether textual indicators derived from free‐recall protocols could reveal aspects of illness script quality in medical students.

**Methods:**

In this cross‐sectional study, students from years one through six at seven Brazilian medical schools completed a free‐recall task for chronic obstructive pulmonary disease (COPD) and myocardial infarction (MI) after a progress test. Written responses were segmented into idea units (IUs) and analysed for accuracy (correct idea units), scope (number of disease dimensions), organisation (balanced adjusted ratio of clustering) and encapsulation (higher‐order summary terms). Differences across training years were examined using Kruskal–Wallis and Dunn tests.

**Results:**

Accuracy and scope increased significantly across training years for both diseases (median correct ideas for COPD: 3 in year 1 versus 14 in year 5, *p* < 0.01; for MI: 8 versus 18, *p* < 0.01). Most gains occurred between years 1–2 and 3, with limited differentiation thereafter. Organisation increased from 0.31 in year 1 to 0.61 in year 5 for COPD and from 0.28 to 0.60 for MI (range −1 to +1; both *p* < 0.01). Encapsulated and incorrect ideas were infrequent at all training levels. Progress test scores correlated positively with the number of correct ideas (COPD rho = 0.56; MI rho = 0.46; *p* < 0.01).

**Conclusions:**

Free‐recall protocols analysed along theoretically grounded dimensions detected differences in accuracy, scope and organisation across training years. Constraints, such as a time‐imposed ceiling effect and limited sensitivity to encapsulated knowledge, should guide future methodological refinement. Despite its preliminary nature, this methodology may contribute to research on clinical expertise by offering a novel approach to studying illness scripts across training levels.

## Abstract 2

Can we track students' development of illness scripts through the analysis of recall protocols? Read on to find out. #MedEd

## 1 INTRODUCTION

Diagnostic expertise is a pillar of medical practice, forming the backbone of safe patient care. Consequently, understanding the mechanisms involved in diagnostic expertise development is of paramount importance for educators, as they strive to support this complex process through educational interventions. However, despite significant advances in identifying the cognitive processes underlying diagnostic reasoning,^1^ a detailed understanding of how this expertise develops over time and its critical transition points during medical training remain incomplete. This insufficient understanding limits our ability to craft educational strategies that are precisely aligned with learners' needs.

### 1.1 Theoretical framework

During the 1970s, Elstein and colleagues found that both physicians and medical students used similar cognitive processes, generating diagnostic hypotheses early in the patient encounter by recognising a few sets of symptoms and signs.^2^ This suggested that expertise is not related to differences in cognitive strategies but to the quality of the hypotheses generated in the recognition step. This step can be understood as a largely unconscious match of clinical data with mental representations of disease knowledge stored in memory.^1^ This observation led to a shift in research emphasis towards exploring how knowledge is developed and organised.^3^

In the following decade, Norman, Schmidt and others^4^, ^5^ understood that expertise follows the development of more nuanced and robust mental representations of diseases, which are related to increased diagnostic accuracy. The creation of such mental representations starts with the elaboration of simple mechanistic explanations of symptoms and signs that evolve into complex semantic networks enriched with nuances accumulated through the exposure to real patients.^6^ These complex representations are called illness scripts and have three main components: (a) fault, which is the disease's pathophysiology; (b) enabling conditions, which are the personal and contextual attributes that make a particular patient susceptible to that disease; and (c) consequences, which are the clinical manifestations of the disease.^1^ Illness scripts play a crucial role in the diagnostic process by allowing clinicians to recognise and interpret patterns of clinical information, matching them with stored knowledge.^1^

Empirical studies demonstrated that script components are processed differently across expertise levels. Custers, Boshuizen and Schmidt^7^ demonstrated that experts and novices differ not only in the amount of knowledge they bring to a case, but in how illness script components influence their processing of clinical information and probability estimates. Experts tend to use more enabling conditions, and their knowledge is encapsulated; that is, they use higher‐order medical terms with a large explaining power. Similarly, studies using recall and think‐aloud protocols have shown that the relationship between expertise and the use of biomedical knowledge follows a nonlinear pattern, with intermediates producing more elaborate pathophysiological explanations than both novices and experts, a finding consistent with the encapsulation hypothesis.^3^, ^8^, ^9^ However, although these studies have illuminated the characteristics of expert knowledge, how illness scripts are structured in students at successive stages of training (and what components may be underdeveloped) remain largely unexplored.

### 1.2 From theory to educational practice

Recently, the rise of the patient safety movement^10^ and the recognition of knowledge as the protagonist in diagnostic error reduction^11^ brought renewed interest in the research on illness scripts. This revival includes a call for applying this theoretical understanding of expertise development into the creation of meaningful pedagogical activities, capable of scaffolding the development of such mental representations.^8^ For instance, it is likely that students who are in different stages of illness script development would benefit most from different educational strategies.^12^, ^13^ To tailor such strategies, educators must have tools to gain insight into and track students' illness script quality and development. However, these tools are presently lacking. The Script Concordance Test (SCT), for example, assesses the degree to which learners' clinical judgements align with expert opinion under conditions of uncertainty.^14^, ^15^ Although the SCT has demonstrated sensitivity to differences across training levels, it evaluates concordance with expert reasoning rather than the underlying structure of illness scripts themselves. Well‐established methods for assessing knowledge, from multiple choice questions to essays and simulations, also exist. Nevertheless, they cannot inform teachers on the accuracy, scope and organisation of their students' illness scripts, and gaps requiring improvement may be overlooked.

### 1.3 Study purpose and expectations

Therefore, this study explores a methodology grounded in cognitive psychology research (specifically, free‐recall tasks) to examine written expressions of disease knowledge as indirect proxies for students' illness scripts. We analysed the scope, accuracy, organisation and encapsulation of information presented in students' free‐recall texts. We anticipated accuracy to rise (and errors to fall) steadily from year 1 to 6; scope to expand sharply in years 1–3 before plateauing (with ‘treatment’ peaking late); organisation to improve rapidly once case‐based learning and patient contact begin (years 3 and 4); and encapsulation to be minimal in the basic‐science phase but increase during clerkships.

## 2 METHODS

### 2.1 Design, participants and context

This research was designed as an exploratory cross‐sectional observational study. All (3457) students from seven Brazilian medical schools from all years of training were invited to participate in October 2022.

In Brazil, students enrol in medical school right after high school. All seven participating schools follow the Brazilian national guidelines for medical education, which mandate a 6‐year programme divided into three phases^16^: *Years 1 and 2 (Basic Cycle)* – integrated basic sciences and introductory clinical skills; *Years 3 and 4 (Clinical Cycle)* – organ‐system courses delivered in problem‐based or case‐based formats, combined with *early patient care* in internal medicine, surgery, paediatrics, obstetrics–gynaecology and family medicine; *Years 5 and 6 (Clerkship)* – full‐time clinical rotations in the aforementioned specialties plus emergency medicine and community health.

The study was performed during the 2022 application of an annual progress test, which is described in detail on the Supporting Information Digital Content, along with more information on the schools' curricula. Participation was optional, and informed consent was obtained. The study was approved by the local university research ethics committee (CAAE 60683822.4.1001.5411).

### 2.2 Procedures

The seven schools run a single Progress Test (PT), administered to all students in all years of training, run online at the same date and time.^17^ The PT was conducted remotely, with technical support, and facilitated the collection of data, allowing the recruitment of all students simultaneously. The PT consisted of 120 multiple‐choice items and lasted approximately 4 hours. Participation in the recall task was voluntary and occurred immediately after the test. Students were invited by faculty members at their respective schools. No academic credit was offered for participation. An invitation with a weblink to participate in the study was included in the final screen of the PT. The link forwarded participants to Harena,^18^ an online platform that collected participants' data, including year of medical training, school of origin and the answers to our recall task.

### 2.3 Recall task

Free recall is a well‐established task in cognitive psychology for studying how knowledge is organised and retrieved from long‐term memory.^19^ In medical education research, recall tasks have been used with different instructions depending on the research focus: studies on the intermediate effect and encapsulation typically asked participants to process and then recall case information,^3^, ^5^ whereas studies focused on enabling conditions asked participants to describe the prototypical clinical picture of a disease.^6^ In the present study, rather than presenting a clinical case or targeting a specific script component, we asked the participants to type everything they knew about a named disease, seeking to capture multiple dimensions simultaneously (accuracy, scope, organisation and encapsulation) from a single open‐ended prompt. Tulving and Pearlstone^19^ showed that information stored in memory is not always accessible through free recall: what can be retrieved depends on the cues available at the time of retrieval. Drawing on this distinction between availability (knowledge stored in memory) and accessibility (knowledge that can be retrieved under specific conditions), our rationale was that the way disease knowledge is retrieved in response to an open‐ended prompt may provide indirect information about the underlying organisation of illness scripts.

In this study, the free‐recall task consisted of students being instructed to ‘type everything you know about myocardial infarction’ and then to do the same for chronic obstructive pulmonary disease, with a 5‐minute time limit for each. No additional thinking time was given. We chose the 5‐minute cap after pilot testing with practicing physicians, who were able to produce concise yet substantively complete responses in that time frame. A review of the student protocols confirmed that many learners likewise delivered rich, well‐organised answers, indicating that the limit was workable. These two diseases were selected because they are prevalent, clinically important and covered from early years in all participating curricula, ensuring that students at all training levels could produce meaningful responses.

### 2.4 Illness scripts dimensions

Students' recall protocols with the descriptions of the diseases were segmented into idea units (IUs), the smallest meaningful fragments of text. For example, ‘Chest pain radiating to the jaw’ contains four IUs of increasing specificity (pain; chest pain; chest pain radiating; chest pain radiating to the jaw), each meaningful for differentiating response quality. We expected novice learners to generate many simple, discrete IUs, while more advanced trainees would condense them into higher‐order concepts, producing denser and more hierarchical units. Idea units could also be built from non‐adjacent pieces of text. For example, in the sentence ‘Spirometry in COPD patients is a very important test and usually shows an obstructive defect with a reduced FEV1/FVC’, one of the IUs is ‘spirometry + obstructive defect’.

Each idea unit was classified into one or more disease dimensions: pathophysiology, epidemiology, aetiology, history, physical examination, complementary exams, differential diagnosis and therapeutic plan. Our eight dimensions align with the canonical components of illness scripts as follows: *enabling conditions* (epidemiology, relevant history), *fault* (pathophysiology, aetiology) and *consequences* (history findings, physical‐examination signs, complementary exams, differential diagnoses). Although not part of the original illness script triad,^1^ the therapeutic plan was retained as a separate dimension given its clinical importance and underrepresentation in clinical reasoning research. IUs were further classified as correct or incorrect and as encapsulated or simple. One example of an encapsulated IU in MI responses is ‘atherosclerosis’: one word with the same explanatory power as several sentences that could be used to describe the process of plaque formation in arteries.

We examined four components: accuracy (number of correct idea units), scope (number of distinct disease dimensions), organisation (level of clustering via the Adjusted Ratio of Clustering, explained in the succeeding text), and encapsulation (number of higher‐order summary terms). Examples of the scoring procedure are provided in Figures S1–S4.

We analysed the organisation using the Adjusted Ratio of Clustering (ARC),^20^, ^21^ which measures how closely idea units from the same dimension cluster together in a text, previously applied in clinical reasoning research.^5^ ARC ranges from any negative value, through zero (chance‐level clustering), to +1 (perfect clustering). Because ARC does not account for the number of disease dimensions covered, we also computed a balanced ARC = ARC × log10(*C*), where *C* is the number of distinct dimensions mentioned.^22^ This adjustment rewards breadth sublinearly, keeping the score on a more conventional −1 to +1 scale. Further details and rationale are provided in Supporting Information Digital Content.

### 2.5 Blueprint creation and reliability

Firstly, through initial analyses from a sample of participants' responses, we developed a blueprint with general and disease‐specific rules and recommendations for the identification of IUs.^23^ The blueprint was developed by three authors with complementary expertise in clinical medicine, medical education and research in clinical reasoning.

To minimise individual bias, we began with a consensus exercise. The junior author (FSV) first coded a set of pilot responses, which were then reviewed in team meetings with MACF and SM. Through successive rounds of discussion, the team calibrated the desired level of granularity and compiled the resulting decisions into a written blueprint containing general coding rules (e.g. that idea units could be formed from non‐adjacent text segments), disease‐specific reference lists of expected encapsulated terms and worked examples. Correct versus incorrect labels were assigned with reference to current international guidelines and standard textbooks. The blueprint was also reviewed by a different member of the research team (LMCR).

After the consensus blueprint was finalised, and before independent coding began, the junior author conducted a calibration session with the second coder (HR), demonstrating the annotation procedure and reviewing the blueprint rules. The two coders independently evaluated 20% of the sample. Inter‐rater reliability was evaluated with a two‐way random, absolute‐agreement ICC,^24^ appropriate for count data that can take >2 values. Reliability was evaluated on a subset of the data to confirm the consistency of the coding framework before applying it to the remaining material.^25^, ^26^ We then proceeded with coding by a single rater (FSV) for the remaining 80% of the dataset.

The blueprint, including coding rules, disease‐specific reference lists and worked examples, is available from the corresponding author upon reasonable request.

### 2.6 Statistical analyses

The number of correct/incorrect IU and encapsulated IU, the number of categories mentioned, the ARC and the balanced ARC were summarised as medians by year of training and compared between the six groups (six undergraduate years). Several of our variables violated equal‐variance or residual normality assumptions required for analysis of variance (ANOVA), so we employed the Kruskal–Wallis test to determine if there were statistically significant differences between the groups. To identify which specific groups differed from each other, post hoc pairwise comparisons were conducted using Dunn's test with a Bonferroni correction for multiple comparisons. Spearman rank correlations were computed between progress test scores and the number of correct IUs as a complementary analysis. All analyses were performed using R Statistical Software (version 4.3.1; R Core Team 2023). Significance level was set at *p* < 0.05.

## 3 RESULTS

A total of 435 chronic obstructive pulmonary disease (COPD) COPD and 451 myocardial infarction (MI) recall protocols were collected. After removing blank and nonsensical texts, 429 COPD and 447 MI recall protocols were analysed. Number of students from years 1 to 6 were, respectively: 46, 48, 69, 90, 98 and 78 (total 429; response rate 12%) for COPD, and 55, 52, 69, 92, 99 and 80 (total 447; response rate 13%) for MI.

### 3.1 Interrater reliability

Values for ICC for all variables were as follows: COPD correct ideas 0.95, MI correct ideas 0.93; COPD dimensions 0.91, MI dimensions 0.83; COPD encapsulated ideas 0.57; COPD incorrect ideas 0.57; and COPD ARC 0.50, MI ARC 0.78. ICCs were low for MI incorrect and encapsulated ideas (0.12 and 0.23, respectively), indicating that inter‐rater reliability for these variables did not differ significantly from chance. These variables also had very low base rates (medians of 0 across most years), which limits both the reliability and the interpretability of group comparisons. ICC values above 0.75 indicate excellent agreement, 0.60–0.74 good, 0.40–0.59 fair and below 0.40 poor.^27^ Accordingly, agreement was excellent for correct ideas and COPD dimensions, good for MI dimensions and COPD encapsulated ideas, and poor for MI incorrect and encapsulated ideas. A detailed breakdown of agreement patterns between the two coders is provided in Table S1.

### 3.2 Task time

There was a monotonic increase in time spent across training years for both COPD (Jonckheere–Terpstra [JT] = 49.33, *p* < 0.01) and MI (JT = 52.28, *p* < 0.01) responses. From year 3 onwards, the median response time approached the 5‐minute limit, suggesting that more advanced students were increasingly constrained by the time cap. Detailed time‐use distributions by training year are provided in Figures S5 and S6.

### 3.3 Accuracy and encapsulation

The number of correct idea units increased across training years for both diseases (Figure 1). For COPD, the median rose from 3 (IQR 2–7) in Y1 to 10 (8–14) in Y3 and continued to increase more gradually to 14 (11–19) in Y5, remaining stable in Y6 (14 [11–17.8]). For MI, a similar pattern was observed: the median rose from 8 [5–11] in Y1 to 14 [12–18] in Y3, reaching 18 [13.5–21.5] in Y5 before stabilising in Y6 (17 [12–21]). For both diseases, the most pronounced gains occurred between Y1–2 and Y3, coinciding with the transition to clinical training. Post hoc comparisons confirmed significant differences between Y1–2 and all subsequent years, with few significant differences among Y3–6 (Table 1).

**FIGURE 1:**
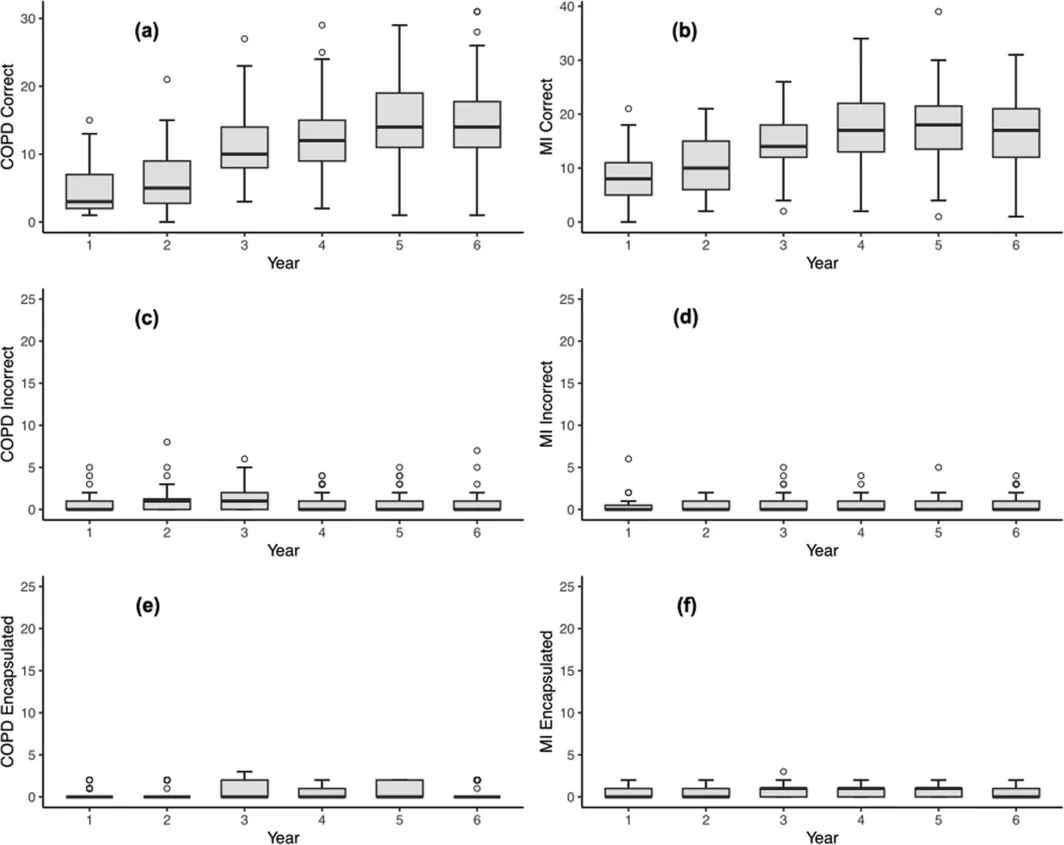
Distribution of idea unit counts by training year. Boxes show median (horizontal line) and interquartile range (box edges); whiskers extend to 1.5 × IQR; circles represent values beyond 1.5 × IQR. (a) Correct ideas, COPD. (b) Correct ideas, MI. (c) Incorrect ideas, COPD. (d) Incorrect ideas, MI. (e) Encapsulated ideas, COPD. (f) Encapsulated ideas, MI. COPD, chronic obstructive pulmonary disease; IQR, interquartile range; MI, myocardial infarction.

**TABLE 1:** Accuracy, scope, organisation and encapsulation indicators (median [IQR]) by training year for COPD and MI.

Disease	Variable	Y1	Y2	Y3	Y4	Y5	Y6	*p* ^a^	Pairwise summary^b^
MI	Correct ideas	8 [5–11]	10 [6–15]	14 [12–18]	17 [13–22]	18 [14–22]	17 [12–21]	<0.01	Y1–2 < Y3–6
MI	Incorrect ideas	0 [0–1]	0 [0–1]	0 [0–1]	0 [0–1]	0 [0–1]	0 [0–1]	0.26	n.s.
MI	Encapsulated ideas	0 [0–1]	0 [0–1]	1 [0–1]	1 [0–1]	1 [0–1]	0 [0–1]	0.01	Y1 < Y3–5
MI	Dimensions	3 [1–3]	3 [2–4]	4 [3–5]	4 [4–5]	4 [4–5]	4 [4–5]	<0.01	Y1–2 < Y3–6
MI	ARC	0.59 [0.00–0.91]	0.78 [0.52–1.00]	0.90 [0.82–1.00]	0.88 [0.71–1.00]	0.91 [0.82–1.00]	1.00 [0.82–1.00]	<0.01	Y1 < Y3–6; Y2 < Y6
MI	Balanced ARC	0.28 [0.00–0.41]	0.34 [0.24–0.51]	0.52 [0.40–0.63]	0.55 [0.43–0.62]	0.57 [0.48–0.64]	0.60 [0.48–0.66]	<0.01	Y1–2 < Y3–6
COPD	Correct ideas	3 [2–7]	5 [3–9]	10 [8–14]	12 [9–15]	14 [11–19]	14 [11–18]	<0.01	Y1–2 < Y3–6; Y3 < Y5
COPD	Incorrect ideas	0 [0–1]	1 [0–1]	1 [0–2]	0 [0–1]	0 [0–1]	0 [0–1]	<0.01	Y3 > Y6
COPD	Encapsulated ideas	0 [0–0]	0 [0–0]	0 [0–2]	0 [0–1]	0 [0–2]	0 [0–0]	<0.01	Y1–2 < Y3; Y3 > Y6
COPD	Dimensions	2 [1–3]	3 [2–4]	4 [4–5]	5 [4–6]	6 [5–6]	5 [4–6]	<0.01	Y1–2 < Y3–6; Y3 < Y5
COPD	ARC^c^	0.82 [0.53–1.00]	1.00 [0.56–1.00]	0.73 [0.53–0.89]	0.85 [0.66–1.00]	0.84 [0.72–1.00]	0.80 [0.72–0.94]	0.046	Y3 < Y5
COPD	Balanced ARC^c^	0.31 [0.28–0.48]	0.48 [0.30–0.51]	0.46 [0.34–0.60]	0.55 [0.43–0.67]	0.61 [0.50–0.70]	0.60 [0.48–0.62]	<0.01	Y1–2 < Y4–6; Y3 < Y4–6

*Note*: Values are median [interquartile range]. Y1–Y6 = training years 1–6. n.s. = not significant.

Abbreviations: ARC, adjusted ratio clustering; COPD, chronic obstructive pulmonary disease; IQR, interquartile range; MI, myocardial infarction.

^a^ Kruskal–Wallis test.

^b^ Significant pairwise differences (*p*< 0.05, Bonferroni‐corrected Dunn test).

^c^ COPD ARC: *n* = 390 (39 single‐dimension texts excluded); all other COPD: *n* = 429; MI: *n* = 447.

Incorrect ideas were rare: the median was 0 (IQR 0–1) across most training years for both diseases, with upper quartiles not exceeding 2 for COPD and 1 for MI. There was no significant difference across years for MI (*p* = 0.26), and there was a small difference for COPD (*p* < 0.01), driven primarily by a higher median in Y3 (1 [0–2]) compared to Y6 (0 [0–1]). Encapsulated ideas were similarly infrequent: for COPD, the median was 0 across all years, with upper quartiles ranging from 0 to 2; for MI, the median was 0 in Y1–2 and Y6 and 1 in Y3–5, with upper quartiles of 1 throughout. Although statistically significant differences were found (COPD and MI *p* < 0.01), the absolute values were too low to support meaningful interpretation.

### 3.4 Scope and organisation

The number of disease dimensions represented in participants' responses increased across training years (Figure 2). For COPD, the median rose from 2 (IQR 1–3) in Y1 to 4 (4–5) in Y3 and 6 (5–6) in Y5. For MI, the median rose from 3 (1–3) in Y1 to 4 (3–5) in Y3, remaining at 4 (4–5) in Y5 and Y6. As with accuracy, the sharpest gains occurred between Y1–2 and Y3.

**FIGURE 2:**
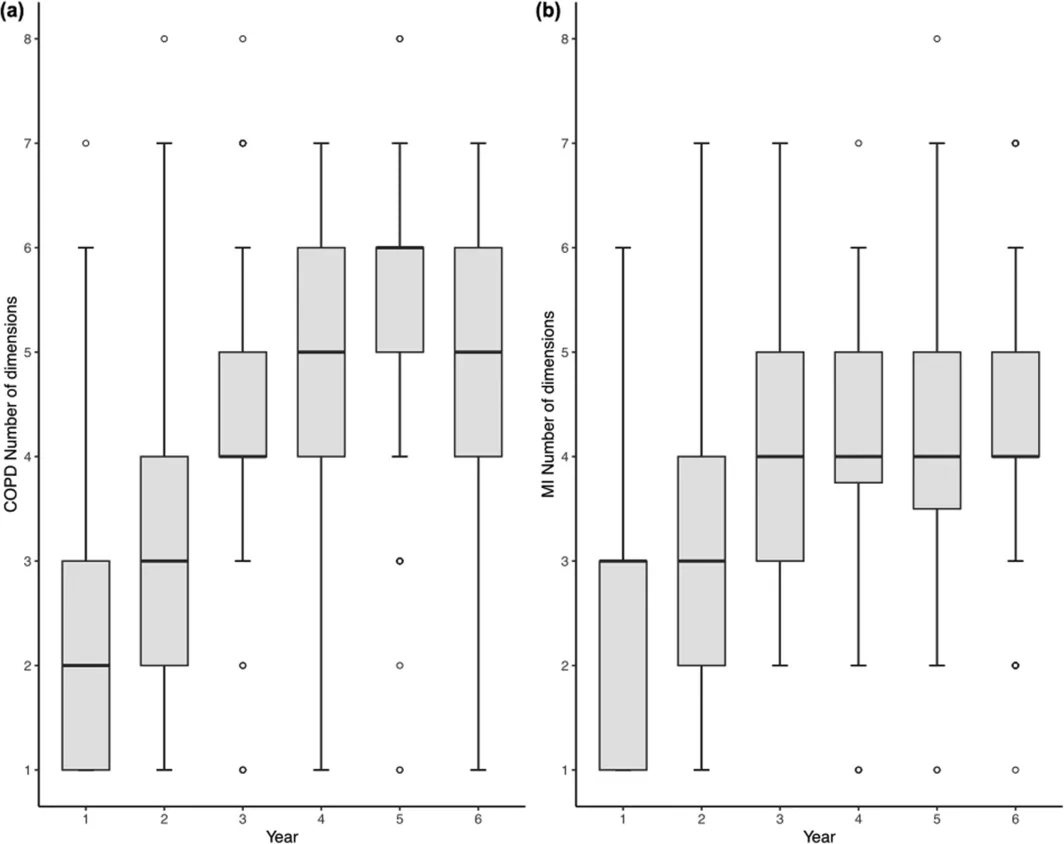
Number of disease dimensions per response, by training year. Boxes show median and interquartile range; whiskers extend to 1.5 × IQR; circles represent outliers. (a) COPD. (b) MI. COPD, chronic obstructive pulmonary disease; IQR, interquartile range; MI, myocardial infarction.

Organisation, measured by the ARC, showed a clearer pattern for MI than for COPD (Figure 3). For MI, the median ARC increased from 0.586 [0.000–0.907] in Y1 to 0.903 [0.815–1.000] in Y3, reaching 1.000 [0.823–1.000] in Y6. Post hoc comparisons showed significant differences between Y1 and all years from Y3 onwards. For COPD, the pattern was less distinct: the median ARC was already 0.820 in Y1, although this estimate is based on only 30 students (16 of the 46 Y1 participants were excluded because single‐dimension texts yield undefined ARC values). The overall Kruskal–Wallis test was marginally significant (*p* = 0.046).

**FIGURE 3:**
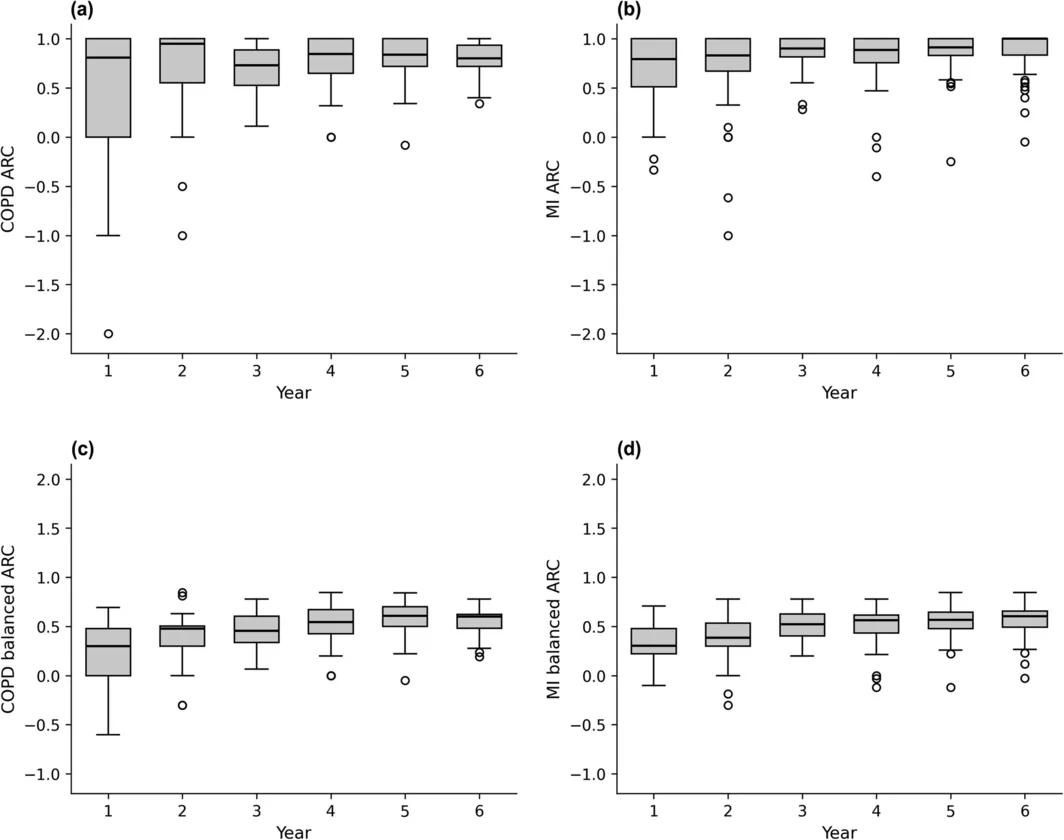
Adjusted ratio of clustering (ARC) by training year. ARC measures how closely idea units from the same dimension cluster together (range: any negative value to +1). (a) ARC, COPD. (b) ARC, MI. (c and d) Balanced ARC, which applies a logarithmic adjustment for the number of dimensions represented (range: any negative value to +1). (c) Balanced ARC, COPD. (d) Balanced ARC, MI. Boxes show median and interquartile range; whiskers extend to 1.5 × IQR; circles represent outliers. COPD, chronic obstructive pulmonary disease; IQR, interquartile range; MI, myocardial infarction.

The balanced ARC, which accounts for the number of dimensions represented, showed a more consistent pattern across both diseases. For COPD, the median increased from 0.312 (0.276–0.477) in Y1 to 0.608 (0.499–0.699) in Y5 (*p* < 0.01). For MI, it increased from 0.280 (0.000–0.414) in Y1 to 0.602 (0.484–0.657) in Y6 (*p* < 0.01). Significant pairwise differences were found between Y1–2 and Y4–6 for both diseases (Table 1).

### 3.5 Progress test performance

Progress test scores increased from the first to the sixth year with a seemingly constant gradient (Figure S7; Kruskal–Wallis, *p* < 0.01). There was a positive linear correlation between the test scores and the number of correct ideas for both COPD (Spearman rho = 0.56, *p* < 0.01) and MI (rho = 0.46, *p* < 0.01).

## 4 DISCUSSION

This study examined whether textual indicators derived from free‐recall responses about COPD and MI could shed light on illness script quality across 6 years of medical training. We analysed the responses along the dimensions of accuracy, scope, organisation and encapsulation. Three main findings emerged. First, we observed that accuracy and scope increased significantly between the first and third years, with most pairwise differences concentrating in the transition from preclinical to clinical training. Second, organisation, measured by the balanced ARC, followed a similar pattern, with the sharpest gains in the early years. Third, contrary to our expectations, we noticed that encapsulated ideas were infrequent at all training levels and did not increase over time, and incorrect ideas were rare throughout.

Our findings suggest that these indicators may provide a useful, albeit indirect, way to explore differences in students' illness scripts across training levels. At the same time, as with any study that infers mental representations from observable performance, our approach is subject to inherent constraints. The 5‐minute time limit may have imposed a ceiling on more advanced students, the open‐ended prompt may have favoured breadth over encapsulation and the inter‐rater reliability was low for some variables. These are common challenges in research that uses free‐recall and text‐based protocols to study knowledge organisation.^28^, ^29^

### 4.1 Scope and accuracy

As expected, we observed that both rose sharply between the second and third years, when clinical exposure intensifies. Contrary to our expectations, we observed a very low number of incorrect IUs. This pattern is consistent with the theoretical expectation that, as learners accumulate clinical experience, their illness scripts become richer in accurate information. However, the low number of incorrect ideas may also reflect students filtering uncertain content under time pressure, rather than an absence of misconceptions.

### 4.2 Organisation

Our findings suggest that it is possible to analyse and investigate the organisation of medical students' illness scripts, and that the differences in organisation across training years parallel the differences in knowledge breadth. However, we did not observe a continuing increase in organisation after the middle of the undergraduate training, which can be at least partially explained by a ceiling effect (students quickly achieved ARC values close to the maximum of one).

### 4.3 Encapsulation

The number of encapsulated ideas did not grow over the years of training. The instruction to ‘type everything you know’ may have led students to prioritise breadth over depth, potentially reducing their use of concise, encapsulated terms. This is consistent with the encoding specificity principle^30^: encapsulated knowledge is likely formed through clinical encounters, and a prompt that names the disease without presenting a clinical scenario may not provide the retrieval conditions needed to access such knowledge. Moreover, van de Wiel et al.^31^ showed that when explaining clinical concepts, even experienced participants tend to unpack higher‐level encapsulated knowledge into its constituting elements, a process that our open‐ended prompt may have similarly encouraged and that contrasts with findings from studies using case‐based recall.^32^, ^33^

## 5 FUTURE DIRECTIONS

This study represents an initial step in a broader research programme aimed at developing theoretically grounded methods to study illness script quality. Our findings highlight several methodological questions that warrant further investigation.

In our view, one important direction is understanding how task design shapes the indicators we proposed. Our prompt proved sensitive to differences in accuracy, scope and organisation. Whether alternative task formats, such as case‐based or explanation‐oriented prompts, would additionally capture dimensions like encapsulation is an open empirical question worth exploring in future studies, potentially in comparison with the present approach. A second priority is extending this approach to more experienced participants. Including practicing physicians would provide a reference point against which student patterns can be compared and could help clarify whether the levelling‐off we observed reflects a genuine feature of expertise development or is an artefact of our task design. Third, extending this approach to a broader range of diseases (including less prevalent conditions and those with different pathophysiological complexity) is an important direction for future research, as is exploring different settings and raters. Future studies could also examine whether curricular differences across schools influence the observed patterns.

Scalability is a central consideration for the future of this approach. The development of a disease‐specific blueprint requires iterative consensus among experienced clinician–researchers, and the manual coding of over 800 protocols required considerable time and expertise. Addressing the methodological limitations identified in this study is an important step before broader implementation. Looking ahead, blueprints could be shared across institutions, potentially building a collaborative database similar to shared item banks for progress tests. Also, recent advances in large language models may help reduce the coding burden. Our research group is working on LLM‐based annotations, and we plan to test the reliability with human coding across the dimensions examined in this study. The theoretically grounded framework developed in the present study may offer the conceptual basis upon which such tools can be built and evaluated.

## 6 LIMITATIONS

Our study has important limitations. First, the 5‐minute time restriction may have introduced construct‐irrelevant variance to the data.^34^ It may have imposed a ceiling that attenuated differences among more advanced learners. The apparent levelling‐off in clinical years may reflect task constraints rather than a true plateau. Also, because performance under these time‐constrained conditions may depend on retrieval fluency or efficiency, the task may have captured a construct partly distinct from the one we originally intended to evaluate. Second, students' motivation and effort may have influenced responses; COPD had fewer respondents and more single‐dimension texts, suggesting fatigue. The overall response rate (~12%) is an additional limitation. Third, the open‐ended prompt may have elicited broader responses than we anticipated. The resulting texts should be interpreted as indirect proxies for illness scripts, shaped by both the underlying knowledge structure and the constraints of the task. This may also help explain the low counts of encapsulated ideas and incorrect ideas. Future studies should test alternative prompts designed to elicit more clinically integrated and encapsulated knowledge. Fourth, after establishing inter‐rater reliability on 20% of the dataset, the remaining protocols were coded by a single rater. Although this is consistent with standard practice in text analysis^25^, ^26^ and cognitive research on clinical reasoning,^6^, ^28^, ^29^ the approach does not allow us to assess potential coder drift, and replication with independent coders would strengthen confidence in the findings. Fifth, inter‐rater reliability varied across the coded variables. Although it was high (ICC = 0.83–0.95) for important measures (namely the number of correct ideas and the number of dimensions), it was more modest for variables requiring greater interpretive judgement and that were infrequent in the dataset, such as the encapsulated and incorrect ideas. For all these reasons, findings related to variables with lower reliability should be interpreted with caution. Additionally, the balanced ARC was introduced as an exploratory metric to account for the number of dimensions used in the responses; formal evaluation of its psychometric properties should be undertaken in future work. Finally, our illness‐script blueprint was developed by a core team of clinician–researchers, which can raise questions about broader applicability, even though the blueprint was informed by the team's combined clinical and research backgrounds. It is also important to emphasise that this is an exploratory and not a validation study of the proposed methodology. We did not aim to establish its psychometric properties, and further research should focus on systematically evaluating its reliability and validity before widespread implementation.

## 7 CONCLUSIONS

This study introduces a novel, empirically grounded method for studying illness scripts through free‐recall protocols and textual analysis. Despite the preliminary nature of our findings and methodological limitations, the approach was able to detect differences in accuracy, scope and organisation across training years, warranting further investigation.

## AUTHOR CONTRIBUTIONS

**Fernando Salvetti Valente:** Conceptualization; investigation; writing—original draft; methodology; writing—review and editing; formal analysis; data curation. **Ligia Maria Cayres Ribeiro:** Investigation; writing—review and editing; methodology; supervision; data curation. **Andre Santanchè:** Software; methodology; visualization; writing—review and editing; data curation. **Hassan Rahhal:** Writing—review and editing; formal analysis; investigation; data curation. **Silvia Mamede:** Conceptualization; investigation; methodology; writing—review and editing; supervision; data curation. **Pedro Tadao Hamamoto Filho:** Writing—review and editing; methodology; investigation; supervision. **Marco Antonio de Carvalho Filho:** Conceptualization; investigation; methodology; writing—review and editing; formal analysis; supervision; data curation.

## ETHICS STATEMENT

This study was approved by the UNESP's Ethics Committee on 6 September 2022. CAAE 60683822.4.1001.5411.

## Supporting information


**Figure S1.** The HARENA online environment with an annotated COPD response.
**Figure S2.** The HARENA online environment with an annotated COPD response.
**Figure S3.** The HARENA online environment with an annotated MI response.
**Figure S4.** The HARENA online environment with an annotated MI response.
**Figure S5.** Time spent in the MI responses.
**Figure S6.** Time spent in the COPD responses.
**Figure S7.** Distribution of progress test performance across training years.

## Data Availability

The data that support the findings of this study are available from the corresponding author upon reasonable request.
