# Supplementary material for: ‘Tell me what you know’: Written recall as a window into illness script quality
Source: Med Educ. 2026 Sep 17;60(12):1392–402. doi: 10.1111/medu.70314 (PMC13736128; doi:10.1111/medu.70314)
Supplement: Supplementary file 1 [file MEDU-60-1392-s001.docx]

Supplemental Digital Content

Table of Contents

[Example texts from the HARENA online environment with annotations	2](#_Toc238739261)

[Details about the annual progress test in Brazil	5](#_Toc238739262)

[Information on medical schools’ curricula	6](#_Toc238739263)

[Balanced ARC: rationale and formula	6](#_Toc238739264)

[Time use in responses	7](#_Toc238739265)

[Distribution of progress test performance across training years	9](#_Toc238739266)

[Agreement patterns between the two coders	9](#_Toc238739267)

[References	10](#_Toc238739268)

# Example texts from the HARENA online environment with annotations

**Figure S1.** The HARENA online environment with an annotated COPD response

**Figure S2.** The HARENA online environment with an annotated COPD response

**Figure S3.** The HARENA online environment with an annotated MI response

**Figure S4.** The HARENA online environment with an annotated MI response

The first step is selecting a section of the text that has been identified as an idea unit. It is possible to select non-adjacent sections in a single idea unit. Then, the buttons on the top left are used to classify that piece of text in: one or more of the disease dimensions; as simple or encapsulated; and as correct or incorrect. On the top right, the system stores the selections. On the bottom right, the number of idea units and the scores are calculated automatically.

# Details about the annual progress test in Brazil

The seven schools involved share a similar curriculum structure, despite some differences on methodological approaches. UEL and UFSCAR use problem-based learning, while the other five have lecture-based curricula – organized either by organs and systems or traditional disciplines. All schools share the same overall organization:

- Two years of basic sciences (e.g., Anatomy, Physiology, Biochemistry - pre-clinical, 40 hours/week).
- Two years of clinical sciences (40 hours/week).
- Two years of clerkship rotations (40 to 60 hours/week).

Similarly to national paradigm shifts, all these schools are changing from a teacher-centered and hospital-based approach to student-centered and community-based education^1^. The Inter-Institutional Center for Studies and Assessment Practices in Medical Education (NIEPAEM) coordinates a common annual Progress Test that all seven schools in our study administer simultaneously. Every year, all students from the first through the sixth year answers the same 120 multiple-choice questions, which are blueprint-mapped to Brazil’s national curricular guidelines and is organized into five disciplinary domains (internal medicine, surgery, pediatrics, obstetrics-gynecology, and public health). Items are written collaboratively by faculty from the member institutions, undergo peer review and central psychometric analysis, and have achieved Cronbach’s alpha values of 0.70 or higher across recent test cycles, including sub-analyses by discipline^2^. After each sitting, students and curriculum committees receive longitudinal score reports that track individual and cohort trajectories. Any procedural changes in the Progress Test are implemented uniformly across all campuses, ensuring year-to-year consistency and inter-institutional comparability^3^.

# Information on medical schools’ curricula

All seven programmes involved in our study are six-year medical courses, and each embeds clinical-reasoning instruction longitudinally, albeit in slightly different ways:

• Integrated (five schools). At UNICAMP, UFSCar, FAMERP, USP-Bauru and UNIFESP, diagnostic reasoning is threaded through problem-based or team-based tutorials, early skills-lab sessions and clerkship case conferences. In these settings students are repeatedly required to name enabling conditions and to justify their differential diagnoses, even though the explicit “illness-script” vocabulary is not used.

• Independent contact hours (two schools). UNESP–Botucatu offers a compulsory Year-3 module entitled Clinical Reasoning that walks students through hypothesis generation, enabling/disabling findings and script organization. UNIFESP also has an elective discipline on clinical reasoning available, though it is not obligatory. UEL schedules final-year clinico-pathological conferences focused on reasoning processes and on articulating enabling conditions when weighing competing hypotheses.

# Balanced ARC: rationale and formula

The standard adjusted ratio of clustering (ARC) rewards texts that keep idea units from the same category together, but it also increases whenever more categories are mentioned; a response covering seven dimensions can appear better organized than one covering two, even if their internal clustering is identical. Multiplying ARC by the raw count of dimensions (ARC × C) over-corrects this problem, as shown by two hypothetical students with similar ARC values (0.65 vs 0.75) but very different breadth (7 vs 3 dimensions): their “balanced” scores would inflate to 4.55 and 2.25, respectively. To minimize this effect, we applied a logarithmic weight: balanced ARC = ARC x log10(C). Because log10(C) rises slowly (1 → 2 → 7 dimensions maps to factors 0 → 0.30 → 0.85), the metric still rewards broader knowledge but prevents disproportionate jumps. The balanced ARC remains on the conventional −1 to +1 range.

# Time use in responses

To test if there was an increase in time spent from the first to the sixth years, we employed the Jonckheere-Terpstra test, which is a non-parametric test used to determine if there is a trend in the data across ordered groups. It tests specifically for monotonic trends rather than simply differences between groups. For the COPD responses, the results were JT = 49,327 with p < 0.001 and for the MI responses, the results were JT = 52,278 with p < 0.001. Both results suggest a monotonic increase in the time spent by participants. The results were visually represented in boxplots as seconds used in responses per year of training (Figures S5 and S6).

**Figure S5.** Time spent in the MI responses

**Figure S6.** Time spent in the COPD responses

# Distribution of progress test performance across training years

**Figure S7.** Distribution of progress test performance across training years

# Agreement patterns between the two coders

Table S1 shows the agreement patterns between the two coders across all variables for 72 double-coded protocols per disease.

**Table S1.** Inter-rater agreement between Coder 1 and Coder 2 for 72 double-coded protocols per disease

| **Variable** | **Disease** | **Coder 1 mean** | **Coder 2 mean** | **Exact agreement** | **Within ±1** | **Within ±2** | **Mean \|diff\|** | **ICC** |
| --- | --- | --- | --- | --- | --- | --- | --- | --- |
| Correct ideas | MI | 13.9 | 13.7 | 9 (12%) | 36 (50%) | 52 (72%) | 2.0 | 0.93 |
|  | COPD | 10.8 | 10.9 | 23 (32%) | 43 (60%) | 58 (81%) | 1.5 | 0.95 |
| Incorrect ideas | MI | 0.7 | 0.8 | 33 (46%) | 59 (82%) | 67 (93%) | 0.9 | 0.12 |
|  | COPD | 0.6 | 1.0 | 36 (50%) | 60 (83%) | 70 (97%) | 0.7 | 0.57 |
| Encapsulated ideas | MI | 0.5 | 0.3 | 40 (56%) | 66 (92%) | 71 (99%) | 0.5 | 0.23 |
|  | COPD | 0.4 | 0.2 | 58 (81%) | 63 (88%) | 72 (100%) | 0.3 | 0.57 |
| Dimensions | MI | 3.7 | 3.4 | 45 (62%) | 67 (93%) | 70 (97%) | 0.5 | 0.83 |
|  | COPD | 4.1 | 3.9 | 38 (53%) | 66 (92%) | 72 (100%) | 0.6 | 0.91 |
| Total IUs | MI | 14.7 | 14.6 | 11 (15%) | 40 (56%) | 55 (76%) | 1.9 | — |
|  | COPD | 11.4 | 11.9 | 26 (36%) | 46 (64%) | 58 (81%) | 1.3 | — |

*72 protocols per disease were independently coded by both coders. Exact agreement = number of protocols where both coders assigned the same count. Within ±1 / ±2 = number of protocols where counts differed by at most 1 or 2. |diff| = mean absolute difference between coders. ICC = intraclass correlation coefficient (two-way mixed, single measures, absolute agreement). Note: for variables with very low base rates (incorrect and encapsulated ideas, where medians were 0 across most years), even small absolute disagreements produce low ICC values despite high rates of exact or near-exact agreement.*

# References

1.	Antunes Dos Santos R, Nunes M do PT. [Medical education in Brazil](https://doi.org/10.1080/0142159X.2019.1636955). Medical Teacher. 2019 Oct;41(10):1106–11.

2.	Hamamoto Filho PT, Hashimoto M, Lima AR de A, Diehl LA, Costa NT, Rehder PM, et al. [Reliability across content areas in progress tests assessing medical knowledge: A Brazilian cross-sectional study with implications for medical education assessments](https://doi.org/10.1590/1516-3180.2023.0291.R1.13052024). Sao Paulo Medical Journal = Revista Paulista De Medicina. 2024;142(6):e2023291.

3.	Filho PTH, Hafner M de LMB, Ribeiro ZMT, Lima AR de A, Diehl LA, Costa NT, et al. [Absence of item origin bias on a Brazilian interinstitutional Progress Test examination: A pooled analysis of items approach](https://doi.org/10.1371/journal.pone.0325734). PLOS ONE. 2025 Jun;20(6):e0325734.
